# The Serological Sciences Network (SeroNet) for COVID-19: Depth and Breadth of Serology Assays and Plans for Assay Harmonization

**DOI:** 10.1128/msphere.00193-22

**Published:** 2022-06-15

**Authors:** Amy B. Karger, James D. Brien, Jayne M. Christen, Santosh Dhakal, Troy J. Kemp, Sabra L. Klein, Ligia A. Pinto, Lakshmanane Premkumar, John D. Roback, Raquel A. Binder, Karl W. Boehme, Suresh Boppana, Carlos Cordon-Cardo, James M. Crawford, John L. Daiss, Alan P. Dupuis, Ana M. Espino, Adolfo Firpo-Betancourt, Catherine Forconi, J. Craig Forrest, Roxie C. Girardin, Douglas A. Granger, Steve W. Granger, Natalie S. Haddad, Christopher D. Heaney, Danielle T. Hunt, Joshua L. Kennedy, Christopher L. King, Florian Krammer, Kate Kruczynski, Joshua LaBaer, F. Eun-Hyung Lee, William T. Lee, Shan-Lu Liu, Gerard Lozanski, Todd Lucas, Damodara Rao Mendu, Ann M. Moormann, Vel Murugan, Nkemakonam C. Okoye, Petraleigh Pantoja, Anne F. Payne, Jin Park, Swetha Pinninti, Amelia K. Pinto, Nora Pisanic, Ji Qiu, Carlos A. Sariol, Viviana Simon, Lusheng Song, Tara L. Steffen, E. Taylor Stone, Linda M. Styer, Mehul S. Suthar, Stefani N. Thomas, Bharat Thyagarajan, Ania Wajnberg, Jennifer L. Yates, Kimia Sobhani

**Affiliations:** a Department of Laboratory Medicine and Pathology, University of Minnesota, Minneapolis, Minnesota, USA; b Department of Molecular Microbiology & Immunology, Saint Louis University, St. Louis, Missouri, USA; c Frederick National Laboratory for Cancer Research, Frederick, Maryland, USA; d W. Harry Feinstone Department of Molecular Microbiology and Immunology, The Johns Hopkins Bloomberg School of Public Health, Baltimore, Maryland, USA; e Department of Microbiology and Immunology, University of North Carolina, Chapel Hill, North Carolina, USA; f Department of Pathology and Laboratory Medicine, grid.471395.dgrid.189967.8 Emory University School of Medicine, Atlanta, Georgia, USA; g Department of Medicine, University of Massachusetts Chan Medical School, Worcester, Massachusetts, USA; h Department of Microbiology & Immunology, University of Arkansas for Medical Sciences, Little Rock, Arkansas, USA; i Department of Pediatrics, University of Alabama at Birmingham, Birmingham, Alabama, USA; j Department of Microbiology, University of Alabama at Birmingham, Birmingham, Alabama, USA; k Department of Pathology, Icahn School of Medicine at Mount Sinai, New York, New York, USA; l Department of Pathology and Laboratory Medicine, Donald and Barbara Zucker School of Medicine at Hofstra/Northwell, Hempstead, New York, USA; m MicroB-plex, Inc., Atlanta, Georgia, USA; n Wadsworth Center, New York State Department of Health, Albany, New York, USA; o Department of Microbiology and Medical Zoology, University of Puerto Rico—Medical Sciences Campus, San Juan, Puerto Rico, USA; p Salimetrics, LLC, Carlsbad, California, USA; q Division of Pulmonary, Allergy, Critical Care and Sleep Medicine, Department of Medicine, grid.471395.dgrid.189967. Emory University, Atlanta, Georgia, USA; r Department of Environmental Health and Engineering, Johns Hopkins Bloomberg School of Public Health, Baltimore, Maryland, USA; s Department of Pediatrics, University of Arkansas for Medical Sciences, Little Rock, Arkansas, USA; t Department of Internal Medicine, University of Arkansas for Medical Sciences, Little Rock, Arkansas, USA; u Arkansas Children’s Research Institute, Little Rock, Arkansas, USA; v Department of Pathology, Case Western Reserve School of Medicine, Cleveland, Ohio, USA; w Department of Microbiology, Icahn School of Medicine at Mount Sinai, New York, New York, USA; x Virginia G. Piper Center for Personalized Diagnostics, Arizona State University Biodesign Institute, Tempe, Arizona, USA; y Department of Biomedical Sciences, School of Public Health, University at Albany, Albany, New York, USA; z Center for Retrovirus Research, The Ohio State University, Columbus, Ohio, USA; aa Department of Veterinary Biosciences, The Ohio State University, Columbus, Ohio, USA; bb Department of Microbial Infection and Immunity, The Ohio State University, Columbus, Ohio, USA; cc Viruses and Emerging Pathogens Program, Infectious Disease Institute, The Ohio State University, Columbus, Ohio, USA; dd Department of Pathology, The Ohio State University Medical Center, Columbus, Ohio, USA; ee Division of Public Health, College of Human Medicine, Michigan State University, East Lansing, Michigan, USA; ff Department of Epidemiology, College of Human Medicine, Michigan State University, East Lansing, Michigan, USA; gg Unit of Comparative Medicine, University of Puerto Rico—Medical Sciences Campus, San Juan, Puerto Rico, USA; hh Department of Internal Medicine, University of Puerto Rico—Medical Sciences Campus, San Juan, Puerto Rico, USA; ii Center for Childhood Infections and Vaccines of Children’s Healthcare of Atlanta, Department of Pediatrics, Emory University School of Medicine, Atlanta, Georgia, USA; jj Department of Microbiology and Immunology, grid.471395.dgrid.189967.8 Emory University, Atlanta, Georgia, USA; kk Emory Vaccine Center, grid.471395.dgrid.189967.8 Emory University, Atlanta, Georgia, USA; ll Yerkes National Primate Research Center, Atlanta, Georgia, USA; mm Department of Medicine, Icahn School of Medicine at Mount Sinai, New York, New York, USA; nn Department of Pathology and Laboratory Medicine, Cedars-Sinai Medical Center, Los Angeles, California, USA; University of Michigan—Ann Arbor

**Keywords:** COVID-19, SeroNet, assay harmonization, serology

## Abstract

In October 2020, the National Cancer Institute (NCI) Serological Sciences Network (SeroNet) was established to study the immune response to COVID-19, and “to develop, validate, improve, and implement serological testing and associated technologies” (https://www.cancer.gov/research/key-initiatives/covid-19/coronavirus-research-initiatives/serological-sciences-network). SeroNet is comprised of 25 participating research institutions partnering with the Frederick National Laboratory for Cancer Research (FNLCR) and the SeroNet Coordinating Center. Since its inception, SeroNet has supported collaborative development and sharing of COVID-19 serological assay procedures and has set forth plans for assay harmonization. To facilitate collaboration and procedure sharing, a detailed survey was sent to collate comprehensive assay details and performance metrics on COVID-19 serological assays within SeroNet. In addition, FNLCR established a protocol to calibrate SeroNet serological assays to reference standards, such as the U.S. severe acute respiratory syndrome coronavirus 2 (SARS-CoV-2) serology standard reference material and first WHO international standard (IS) for anti-SARS-CoV-2 immunoglobulin (20/136), to facilitate harmonization of assay reporting units and cross-comparison of study data. SeroNet institutions reported development of a total of 27 enzyme-linked immunosorbent assay (ELISA) methods, 13 multiplex assays, and 9 neutralization assays and use of 12 different commercial serological methods. FNLCR developed a standardized protocol for SeroNet institutions to calibrate these diverse serological assays to reference standards. In conclusion, SeroNet institutions have established a diverse array of COVID-19 serological assays to study the immune response to SARS-CoV-2 and vaccines. Calibration of SeroNet serological assays to harmonize results reporting will facilitate future pooled data analyses and study cross-comparisons.

**IMPORTANCE** SeroNet institutions have developed or implemented 61 diverse COVID-19 serological assays and are collaboratively working to harmonize these assays using reference materials to establish standardized reporting units. This will facilitate clinical interpretation of serology results and cross-comparison of research data.

## INTRODUCTION

The National Cancer Institute (NCI) Serological Sciences Network for COVID-19 (SeroNet) was launched on 8 October 2020 as a collaborative initiative to expand research on immune responses to severe acute respiratory syndrome coronavirus 2 (SARS-CoV-2). SeroNet is comprised of investigators from 25 U.S. biomedical research institutions, working in partnership with the Frederick National Laboratory for Cancer Research (FNLCR) and the SeroNet Coordinating Center, which is managed by FNLCR (1). Of the 25 participating research institutions, 8 are designated as Serological Sciences Centers of Excellence (funded by U54 grants), 13 are funded with U01 grants to carry out specific research projects related to COVID-19 immunity, and 4 institutions are funded by subcontracts and are designated as Serological Sciences Network Capacity Building Centers (1).

One of the primary goals of this partnership is “to develop, validate, improve, and implement serological testing and associated technologies” (1). To this end, SeroNet formed a working group, the Serology Assays, Samples, and Materials Operations Group (abbreviated as Serology Assay Ops), in December 2020 to allow for coordinated development and collaborative sharing of serology assay procedures and to establish processes for harmonizing and standardizing methodologies using reference materials across institutions. Establishing harmonized and standardized SARS-CoV-2 serological assays can allow cross-comparison and pooling of research study results and facilitate clinical interpretation of results for patient care.

While there are 85 serological assays approved by the FDA for emergency use (2), the quick development of assays has led to the lack of harmonized cutoffs and reporting units. Furthermore, there are no consensus guidelines on reporting standards or clarity on the clinical interpretation and relevance of results. This has created a complex landscape for interpreting both research and clinical serological assay results. For example, several studies have reported on heterogeneity in serological assay performance that would have a significant impact on research study conclusions and clinical interpretations related to longitudinal serosurveillance (3–6). Specifically, certain assays demonstrate reduced sensitivity over time after an initial SARS-CoV-2 infection diagnosis. Muecksch et al. reported that the Abbott SARS-CoV-2 anti-nucleocapsid IgG assay dropped from a peak sensitivity of 98% at 21 to 40 days post-PCR diagnosis to around 70% when patients were tested ≥81 days postdiagnosis, whereas the Roche Elecsys SARS-CoV-2 anti-nucleocapsid total antibody assay and Siemens SARS-CoV-2 anti-receptor-binding domain (anti-RBD) total antibody assay both maintained high sensitivity (95 to 100%) on the same set of serial samples (3). Narowski et al. also found a significant decline in the longitudinal sensitivity of their lab-developed nucleocapsid assay in a study of health care workers (6). Perez-Saez et al. similarly demonstrated that the rates of seroreversion at least 8 months after the initial infection differed greatly depending on the serological assay used (4). While the seroreversion rate of the EuroImmun semiquantitative anti-S1 IgG enzyme-linked immunosorbent assay (ELISA) was 26%, the rates were significantly lower for the Roche anti-nucleocapsid total antibody assay (1.2%) and the Roche semiquantitative anti-RBD total antibody assay (0%) (4). Additionally, numerous studies rely on neutralization assays as gold standard methods for determining the functional relevance of ligand-binding methods, but comparison studies have demonstrated variability in results for live-virus neutralization, pseudovirus neutralization, and surrogate neutralization assays (e.g., ACE2 inhibition assays) (7–9), raising the importance of assay harmonization and standardization across laboratories.

Therefore, SeroNet aims to address these knowledge gaps in SARS-CoV-2 serological assay research by establishing collaborative initiatives to characterize, compare, and harmonize SARS-CoV-2 serological assays. This paper describes the depth and breadth of serological assays developed and implemented within the SeroNet consortium and outlines a proposed process to establish assay traceability to the U.S. SARS-CoV-2 serology standard reference material and to the WHO international standard (IS; 20/136) for these diverse assays, with the ultimate goal of establishing harmonized reporting standards calibrated to the international standard. Availability of both national and international standards is crucial to provide easy accessibility to end users and due to the limited volume of international standard available; all national standards should be calibrated to the international standard to provide harmonized traceability. These collaborative efforts will facilitate cross-comparison of results and provide clarity for their clinical interpretation, including in response to circulating SARS-CoV-2 variants.

## RESULTS

### SeroNet serology assay data.

Of the 25 institutions involved with SeroNet, 23 reported performing between one and seven serology assays and provided descriptive and performance data. Serology assay data were also obtained from the Frederick National Laboratory for Cancer Research (FNLCR) and National Institute of Standards and Technology (NIST), both of which collaborate with SeroNet. Collectively, SeroNet institutions reported development of 27 in-house ELISA methods (Table 1) (6, 10–26). The majority of ELISA methods were developed for testing of serum and/or plasma, with additional methods available for testing dried blood spots (DBS), saliva/oral fluid, and breast milk. Two methods have been granted FDA emergency use authorization (EUA), 3 methods are pending FDA EUA, 4 methods are validated for high-complexity testing in a Clinical Laboratory Improvement Amendments (CLIA)-certified laboratory, and 18 methods are for research use only (RUO). Diagnostic sensitivity and specificity for in-house ELISA methods range from 67.4 to 100% and 90 to 100%, respectively.

**TABLE 1:** Laboratory-developed singleplex ELISAs^*a*^

Sample type(s)	Antigen(s)	Isotype	Result type	Assay sensitivity and specificity	Center/institution	Reference(s)	Regulatory status
Serum, plasma, dried plasma	RBD	IgG (IgA/IgM being eval)	Qualitative	Day 0–7 after infection: sensitivity, 73.01%. Day 8–14 after infection: sensitivity, 100%. Day >15 after infection: sensitivity, 100%; specificity (*n* = 388 samples collected prior to COVID-19 pandemic), 97.68%.	Emory University	21	FDA EUA granted
Serum, plasma	RBD and Spike	IgG, IgM, IgA	Semiquantitative	Sensitivity, 95%; specificity, 100% (*n* = 38 positive, *n* = 74 negative sera tested)	Mount Sinai	12, 19, 20	FDA EUA granted
Serum, plasma, saliva	RBD	Total Ig, with IgG, IgM, IgA titers	Qualitative	Overall sensitivity, 82.5%; overall specificity, 100% (*n* = 300). At >14 days from symptom onset, sensitivity, 100%, and specificity, 100% (*n* = 261).	University of Minnesota	18, 22	Assays validated in a high-complexity-testing CLIA laboratory
Serum, plasma	RBD	IgG, IgM	Qualitative	Sensitivity, 91% for RBD IgG 15–21 days post-onset of symptoms, 100% >21 days post-onset of symptoms, 90% for RBD IgM 15–21 days post-onset of symptoms, and 100% >21 days post-onset of symptoms; specificity, 99.75% for RBD IgG and 100% for RBD IgM	Stanford University	10	Assays validated in a high-complexity-testing CLIA laboratory
Serum, plasma	RBD-ACE2	Total IgG that blocks RBD-ACE2 binding	Semiquantitative	NA; used as a follow-up assay in seropositive specimens	Stanford University	10	Assay validated in a high-complexity-testing CLIA laboratory
Serum, plasma	RBD	IgG, IgM + IgG	Quantitative (IgG); qualitative (IgM + IgG)	Sensitivity, 98% (*n* = 181); specificity, 98.9% (*n* = 181).	University of Puerto Rico	25, 53	Assay validated in a high-complexity-testing CLIA laboratory
Serum, plasma	Spike	IgG	Quantitative	Sensitivity, 98.3% (*n* = 60); specificity, 99.3% (*n* = 150)	Frederick National Laboratory	NR	RUO
Serum, plasma	Spike	IgM	Quantitative	Sensitivity, 93.8% (*n* = 30); specificity, 97.6% (*n* = 80)	Frederick National Laboratory	NR	RUO
Serum, plasma	Nucleocapsid	IgG	Quantitative	Sensitivity, 97% (*n* = 34); specificity, 100% (*n* = 99)	Frederick National Laboratory	NR	RUO
Serum, plasma	Nucleocapsid	IgM	Quantitative	NR	Frederick National Laboratory	NR	RUO
Serum, plasma, saliva	RBD	Total Ig	Qualitative	Sensitivity, 95% (*n* = 259; 9 or more days after symptom onset), specificity, 96% (*n* = 535)	University of North Carolina	6, 16	FDA EUA pending
Serum, plasma, saliva	Spike NTD	Total Ig	Qualitative	Sensitivity, 92% (*n* = 259; 9 or more days after symptom onset), specificity, 94% (*n* = 535)	University of North Carolina	6	FDA EUA pending
Serum	Spike, RBD	IgG	Semiquantitative	NR	CVVR/BIDMC/Harvard	11	RUO
Serum, plasma, breast milk	RBD	IgG, IgA, IgM	Semiquantitative	NR	CVVR/BIDMC/Harvard	14, 23	RUO
Serum, plasma	Spike	IgG	Quantitative	Sensitivity, 100%; specificity, 98.8%	Tulane University	NR	RUO
Serum, plasma	RBD	IgG	Quantitative	NR	Tulane University	NR	RUO
Serum, plasma	Nucleocapsid	IgG	Quantitative	NR	Tulane University	NR	RUO
Plasma, serum	Spike, RBD	IgM, IgG, IgA	Semiquantitative	Spike: IgG, sensitivity, 96.6%, and specificity, 96.7%); IgA, sensitivity, 99.3%, and specificity, 90%; IgM, sensitivity, 97.9%, and specificity, 100%. RBD: IgG, sensitivity, 97.3%, and specificity, 100%; IgA, sensitivity, 99.3%, and specificity, 96.7%; IgM, sensitivity, 97.9%, and specificity, 96.7%. IgG data based on 126 convalescent plasma donors and 30 prepandemic samples; IgM/IgA data based on 20 hospitalized donors and 30 prepandemic samples.	Johns Hopkins University	15	RUO
Serum, plasma	Spike (ECD), RBD	IgG	Semiquantitative	NR	University of Texas-Austin	17	RUO
Serum, plasma	RBD	IgG	Qualitative	Sensitivity, 100% (*n* = 155); specificity, 96.5% (*n* = 133)	Arizona State University	NR	RUO
Serum, DBS	RBD	IgG, IgM	Qualitative	Sensitivity, 97% (*n* = 39); specificity, 100% (*n* = 37)	University of Arkansas for Medical Sciences	54	RUO
Serum, DBS	RBD, spike, nucleocapsid	IgG, IgM	Qualitative	Sensitivity, 97% (*n* = 39); specificity, 100% (*n* = 37)	University of Arkansas for Medical Sciences	13	RUO
Serum, plasma, breast milk	RBD, spike, nucleocapsid	IgG, IgM, IgA	Quantitative (IgG); Qualitative (IgM, IgA)	Sensitivity, 97% (*n* = 114); specificity, 99%	University of Alabama—Birmingham	NR	RUO
Serum, plasma	RBD, nucleocapsid, spike trimer	IgG, IgA	Quantitative	RBD: sensitivity, 70.9% for IgG and 74.4% for IgA; specificity, 100% for both IgG and IgA. Nucleocapsid: sensitivity, 81.4% for IgG and 77.9% for IgA; specificity, 98.5% for IgG and 100% for IgA). Spike trimer: sensitivity, 67.4% for both IgG and IgA; specificity, 98.5% for IgG and 100% for IgA. Data based on PCR-confirmed COVID-19 hospitalized patients (*n* = 86) and negative prepandemic samples (*n* = 65).	University of Massachusetts Chan Medical School	26	RUO
Serum, Plasma	Nucleocapsid	IgG	Qualitative	Sensitivity, 100% (*n* = 44); specificity, 99.5% (*n* = 202)	The Ohio State University	24	FDA EUA pending
Serum	Nucleocapsid	IgG	Qualitative	NR	The Ohio State University	NR	RUO
Oral fluid	Nucleocapsid	IgG	Qualitative	Sensitivity, 92% (*n* = 24); specificity, 98% (*n* = 85)	Salimetrics	NR	RUO

a ACE2, angiotensin-converting enzyme 2; BIDMC, Beth Israel Deaconess Medical Center; CLIA, Clinical Laboratory Improvement Amendments; CVVR, Center for Virology and Vaccine Research; DBS, dried blood spots; ECD, extracellular domain; EUA, emergency use authorization; FDA, Food and Drug Administration; NA, not applicable; NR, not reported; NTD, N-terminal domain; RBD, receptor-binding domain; RUO, research use only.

Eight institutions reported development or use of multiplex or protein arrays for antibody detection (Table 2) (27–37). Sample types include serum, plasma, DBS, saliva, and bronchoalveolar lavage (BAL) fluid. Diagnostic sensitivity and specificity for multiplex and protein array methods range from 85 to 98.8% and 95.2 to 100%, respectively. Neutralization assays were developed by 9 institutions, with sample types including serum, plasma, BAL fluid, nasal wash, DBS, and breast milk (Table 3) (15, 24, 29, 38–50). Assays fall into three mechanistic categories: competitive binding assays, pseudotyped neutralization assays, and live-virus neutralization assays. The competitive binding assay measures the ability of antibodies to block interactions between the SARS-CoV-2 receptor-binding domain and human ACE2 receptor. Virus pseudotype neutralization assays, mainly HIV and vesicular stomatitis virus (VSV) based, use full-length spike incorporated in the viral particle to measure the capability of neutralizing antibodies to block viral entry into the target cells. SARS-CoV-2 live-virus plaque or focus reduction neutralization assays measure the ability of neutralizing antibodies to block the spreading infection of authentic SARS-COV-2 in cell culture. Diagnostic sensitivity and specificity for neutralization methods developed within SeroNet range from 93 to 100% and 97 to 100%, respectively. Lastly, 9 institutions report use of 12 commercial serology methods (Table 4). Commercial methods detect IgG, IgM, and/or total Ig to spike, RBD, and/or nucleocapsid antigens in serum or plasma. Of the commercial methods in use, 10 have FDA EUA, 1 is pending FDA EUA, and 1 is RUO.

**TABLE 2:** Laboratory-developed multiplex assays^*a*^

Sample type(s)	Antigen(s)	Isotype	Result type	Assay sensitivity and specificity	Center/institution	Reference(s)	Regulatory status
DBS, serum	Spike S1, nucleocapsid	IgG	Qualitative	Sensitivity, DBS, 94% for symptomatic (*n* = 774 samples collected >20 days after PCR^+^ result) and 85% for asymptomatic (*n* = 115 samples collected >20 days after PCR^+^ result); specificity, DBS, 99% (*n* = 730), and serum, 99% (*n* = 701)	Wadsworth	27, 28	NYS CLEP approved
Serum, plasma, DBS	Spike, nucleocapsid, RBD	Total Ig	Semiquantitative	Sensitivity, >97%; specificity, 99%	Wadsworth	29	FDA EUA granted; NYS CLEP approved
Serum, plasma, DBS	Spike, nucleocapsid, RBD	IgG, IgM, IgA	Semiquantitative	Sensitivity, >97%; specificity, 99%	Wadsworth	30	NYS CLEP approved; FDA EUA pending
Oral fluid, serum, plasma	Spike, RBD, nucleocapsid	IgG, IgM, IgA	Semiquantitative	Oral fluid IgG assay, sensitivity, 98.8% ≥15 days post-symptom onset (*n* = 81); specificity, 100% (*n* = 127)	Johns Hopkins University, supporting Michigan State University	31, 36	Oral fluid assays validated in a high-complexity-testing CLIA laboratory; serum/plasma RUO
Serum, plasma, BAL, DBS	Spike, RBD (different variants), nucleocapsid	IgG	Quantitative	Sensitivity, >97% (*n* = 89); specificity, 99% (*n* = 260)	Case Western Reserve University	32	RUO
Serum, plasma, saliva, BAL fluid	Spike, RBD, nucleocapsid	IgA	Quantitative	Sensitivity, >98%; specificity, 99%	Case Western Reserve University	32	RUO
Serum, plasma	Spike	IgG	Quantitative	Sensitivity, ≥93%; specificity, 100%	NIST	33	RUO
Serum, plasma	RBD	IgG	Semiquantitative	Sensitivity, ≥93%; specificity, 100%	NIST	33	RUO
Serum, plasma	RBD, nucleocapsid	IgG	Semiquantitative	Nucleocapsid: sensitivity, 90.3% (*n* = 155), and specificity, 98.0% (*n* = 133). RBD: sensitivity, 90.1% (*n* = 155), and specificity, 97.0% (*n* = 133).	Arizona State University	NR	FDA EUA pending
Serum	Spike, nucleocapsid, RBD	IgG, IgM, IgA	Quantitative	NR	Yale	34	RUO
Serum	Alpha, Beta, Gamma, and Delta variants (spike, RBD)	IgG, IgM, IgA	Quantitative	NR	Yale	35	RUO
Saliva	Spike, nucleocapsid, RBD	IgG	Semiquantitative	Sensitivity: nucleocapsid, 97.7%, RBD, 92.9%, and spike, 98.8%. Specificity: nucleocapsid, 95.2%, RBD, 96.4%, and spike, 97.6%. Combined nucleocapsid and spike sensitivity, 96.5%, and specificity, 98.8%.	Salimetrics	NR	RUO
Serum, plasma	Spike S1, S1-RBD, nucleocapsid, S1-NTD	IgG, IgA, IgM (combined); IgG, IgA, IgM (individual)	Quantitative	Sensitivity: combined antigens and isotypes, 99%; S1-RBD combined isotypes, 99%, and S1-RBD IgG, 99%. Specificity: combined antigens and isotypes, 99%, S1-RBD combined isotypes, 99%, and S1-RBD IgG, 99%. During the acute phase, sensitivity, 92%, and specificity 99%.	Emory/MicroB-plex	37	RUO

a BAL, bronchoalveolar lavage; CLIA, Clinical Laboratory Improvement Amendments; NIST, National Institute of Standards and Technology; NYS CLEP, New York State Clinical Laboratory Evaluation Program.

**TABLE 3:** Neutralization assays^*a*^

Sample type(s)	Antibody neutralization assay type	Result type	Assay sensitivity and specificity	Center/institution	Reference(s)	Regulatory status
Serum, plasma, BAL fluid	HIV lentiviral vector	Quantitative	Sensitivity, 100%, and specificity, 100%, using SeroNet FNLCR blinded reference panel set (*n* = 110)	The Ohio State University	24	RUO
Serum, plasma	Live-virus neutralization assay (microneutralization)	Semiquantitative	NR	Mount Sinai	38, 39	RUO
Serum, plasma, BAL fluid	Live-virus neutralization assay (FRNT)	Quantitative	Sensitivity, 93%; specificity, 100%	Saint Louis University	25, 40	RUO
Serum, plasma, BAL fluid	Live-virus neutralization assay (FRNT/FRNT-mNG/PRNT)	Quantitative	NR	Emory	41	RUO
Serum, plasma, DBS	Live-virus neutralization assay (PRNT)	Qualitative	PRNT_50_: sensitivity, 100%; specificity, 97%. PRNT_90_: sensitivity, 97%; specificity, 100%	Wadsworth	29, 42	NYS CLEP approved (serum and plasma)
Serum, plasma, breast milk	VSV pseudotype particle-based assay	Quantitative	NR	University of Alabama—Birmingham	NR	RUO
Serum, plasma, nasal washes	TCID_50_ neutralization assay	Semiquantitative	NR	Johns Hopkins University	15, 43–47	RUO
Serum, plasma	ACE2 competitive binding assay	Quantitative	Sensitivity, 93.8%; specificity, 99.4%	University of Puerto Rico	25	RUO
Serum, plasma	Lentivirus-based pseudovirus assay for Wuhan D614G, Brazil, South Africa, and Delta variants. Assay performed in CHO/ACE2 cells.	Quantitative	Sensitivity, 100%; specificity, 100%	Tulane University	50	RUO

a CHO, Chinese hamster ovary; FNLCR, Frederick National Laboratory for Cancer Research; FRNT, focus reduction neutralization test; HIV, human immunodeficiency virus; mNG, mNeonGreen; PRNT_50_ and PRNT_90_, 50% and 90% plaque reduction neutralization test; TCID_50_, 50% tissue culture infectious dose; VSV, vesicular stomatitis virus.

**TABLE 4:** Commercial assays

Instrument/assay	Antigen(s)	Isotype	Result type	Center/institution	Regulatory status
Abbott Alinity	Spike	IgM	Semiquantitative	Mount Sinai	FDA EUA granted
Abbott Architect	Spike	IgG	Semiquantitative	Cedars-Sinai^*a*^	FDA EUA granted
Abbott Architect	Nucleocapsid	IgG	Qualitative	Cedars-Sinai^*a*^	FDA EUA granted
Beckman Coulter Access	Spike	IgG	Semiquantitative	Arizona State University	FDA EUA granted
Beckman Coulter Access	Spike	IgM	Qualitative	Arizona State University	FDA EUA granted
DiaSorin Liaison	Spike	IgG	Qualitative (Feinstein/Northwell, Kaiser); quantitative (The Ohio State University)	Feinstein/Northwell, Kaiser, The Ohio State University	FDA EUA granted
DiaSorin Liaison	Spike	IgM	Qualitative	Feinstein/Northwell	FDA EUA granted
Kantaro SeroKlir	Spike, RBD	IgG	Semiquantitative	Mount Sinai	FDA EUA granted
Kantaro quantitative SARS-CoV-2	Spike, RBD	IgG	Quantitative	Mount Sinai	FDA EUA pending
Meso Scale Discovery	Spike, nucleocapsid	IgG, IgM	Quantitative	University of Alabama—Birmingham, CVVR/BIDMC/Harvard, Johns Hopkins University, Stanford	RUO
Roche Elecsys anti-SARS-CoV-2	Nucleocapsid	Total Ig	Qualitative	University of Minnesota, Feinstein/Northwell	FDA EUA granted
Roche Elecsys anti-SARS-CoV-2 S	RBD	Total Ig	Semiquantitative	University of Minnesota, Feinstein/Northwell	FDA EUA granted
Siemens Atellica	Spike	Total Ig	Semiquantitative	Kaiser, The Ohio State University	FDA EUA granted

a Samples sent to Abbott Diagnostics for testing.

### Establishment of SeroNet assay traceability to the U.S. SARS-CoV- 2 serology standard and first WHO international standard for anti-SARS-CoV-2 immunoglobulin.

Units for the U.S. SARS-CoV-2 serology standard were initially established by FNLCR based on measurements performed by eight laboratories (Table 5). Subsequently, FNLCR further established traceability of the U.S. SARS-CoV-2 serology standard to WHO IS 20/136 by using four FNLCR ligand binding serology assays, with assessment of neutralization tested at NIAID’s Integrated Research Facility (IRF) (Table 5). The U.S. SARS-CoV-2 serology standard was made available to the public in December 2020. Thus, far, there have been 124 requests for U.S. SARS-CoV-2 standard material and 19 requests for the reference panel samples.

**TABLE 5:** Units assigned to the U.S. SARS-CoV-2 serology standard^*a*^

Units assigned by FNLCR			WHO-calibrated units				
Functional activity	Spike and nucleocapsid IgM	Spike and nucleocapsid IgG	Functional activity	Spike IgG	Nucleocapsid IgG	Spike IgM	Nucleocapsid IgM
200 NU/mL	100 BAU/mL^*b*^	1200 BAU/mL^*b*^	815 IU/mL	764 BAU/mL^*c*^	681 BAU/mL^*c*^	246 BAU/mL^*c*^	1037 BAU/mL^*c*^

a WHO, World Health Organization; NU, neutralizing units; IU, international units.

b BAU/mL, binding assay units per milliliter.

c BAU/mL, binding antibody units per milliliter.

## DISCUSSION

SeroNet has collectively established a diverse array of methodologies for measurement of SARS-CoV-2 antibodies in a variety of biological fluids. Methods include laboratory-developed ELISAs, multiplex assays, and neutralization assays, most used for research-only purposes, as well as commercial assays available for patient care or research studies. Assays have been developed to test unique sample types, including DBS, saliva/oral fluid, breast milk, nasal washes, and bronchoalveolar lavage fluid. Binding assays identify IgM, IgG, IgA, and/or total antibodies to nucleocapsid, spike, RBD, and/or N-terminal domain (NTD) antigens, and neutralization assays rely on three methods to quantify antibodies with functional neutralizing activity. Assays vary in result reporting, with qualitative, semiquantitative, and quantitative assays. This diversity of assay methods allows for robust investigation of multiple aspects of the serological response to SARS-CoV-2 infection and vaccination and for cross-comparison of assay performance across platforms and institutions within SeroNet.

With the rapid development of numerous methods for serological assessment, as exemplified by the depth and breadth of assays within SeroNet, it is critical to establish assay harmonization and standardized reporting units to facilitate cross-comparison of results across studies, as well as for streamlined meta-analyses. To this end, FNLCR has provided the U.S. SARS-CoV-2 serology standard reference material, which has traceability to the first WHO international standard for anti-SARS-CoV-2 immunoglobulin, to SeroNet sites performing serological assays, to allow establishment of standardized reporting of semiquantitative or quantitative results in binding antibody units (BAU) per milliliter traceable to the WHO standard. For qualitative assays, standardization is crucial for comparing and then harmonizing assay cutoffs for positivity that are traceable to the WHO standard. These efforts may more rapidly facilitate the establishment of a universal cutoff as a correlate of protection, which will be critical to broaden the clinical utility of serological testing for patient care, will allow vaccine trials to transition to an immunogenicity endpoint rather than morbidity or mortality endpoints (immunobridging), and will guide decisions regarding optimal scheduling of future vaccine doses to optimize protective efficacy for the general immunocompetent population and susceptible immunocompromised subpopulations.

While the first step toward harmonization is calibration of assays to a common standard, there will be remaining challenges to pooling data given differences in assay performance metrics, sample types, isotypes, result type (qualitative, semiquantitative, or quantitative), methodologies, and antigen targets. SeroNet and FNLCR continue to work collaboratively to lay the groundwork for effective serology assay data pooling; FNLCR is currently conducting a comprehensive assay comparison study using split blinded samples sent to different SeroNet laboratories to assess the success of harmonization efforts, as well as assay performance (repeatability, sensitivity, and specificity). Currently, there is a broad range of epidemiologic studies being conducted across SeroNet, as previously described, and SeroNet’s future work will include a focus on integrated analysis of pooled data with standardization of reported data elements and assay harmonization (51).

In summary, SeroNet is well positioned to rapidly and collaboratively advance our understanding of the immune response to both SARS-CoV-2 infection and vaccination, with ongoing evaluation of serological responses to SARS-CoV-2 variants of concern. The collective effort of institutions involved with SeroNet, to both establish diverse and complementary serological assays and establish traceability of these diverse assays to the WHO standard, will allow for comprehensive investigation of immune responses and facilitate pooled analyses within the SeroNet consortium. This will enable achievement of the ultimate goal: establishment of a universal correlate-of-protection cutoff, which will provide a foundation for broader clinical use of serological testing, as a guide for future decisions on scheduling of COVID-19 vaccine boosters, as well as for general assessment of COVID-19 vaccine immune responses against vaccine viruses and newly evolving variants of concern.

## MATERIALS AND METHODS

### Compilation of data on SeroNet serological assays.

SeroNet institutions were queried by email between January and July 2021 and asked to complete a comprehensive serological assay survey to describe serological assays developed or implemented at the institutions. The survey requested information on assay and sample type(s), instrument platform and reagents, data output, antibody isotype(s) detected, targeted antigens and virus strain(s), assay performance, cutoffs, use of standards and quality controls, method comparison studies, regulatory status, current use/applications for assays, and publications using each assay.

### Protocol for establishing traceability of serology assays to the U.S. SARS-CoV-2 serology standard and first WHO international standard for anti-SARS-CoV-2 immunoglobulin.

FNLCR developed a protocol for SeroNet institutions to establish serology assay traceability to the U.S. SARS-CoV-2 Serology Standard. Through FNLCR’s participation in the drafting group for the *WHO Manual for the Preparation of Reference Materials for Use as Secondary Standards in Antibody Testing*, the protocol has been made available to the public as of 11 May 2022 (see Appendix 8 of reference 52).

**FIG 1:**
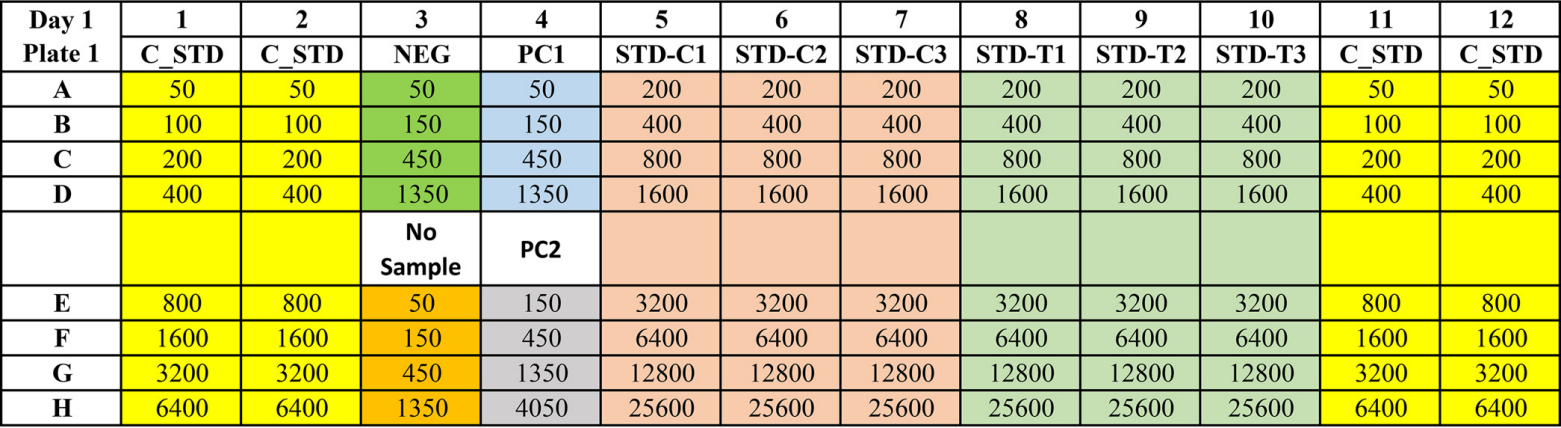
Example plate map for assay calibration setup. Numbers indicate suggested serial dilutions. Serial dilutions of primary and secondary calibrators (reference materials) are plated in triplicate, and the daily internal assay standard is plated in quadruplicate. C_STD, daily internal assay standard; STD-C1, -C2, and -C3, primary calibrator (primary reference material or standard); STD-T1, -T2, and T3, secondary calibrator (secondary reference material or standard); NEG, negative control sample; PC1, positive control sample 1; PC2, positive control sample 2.

In short, for enzyme-linked immunosorbent assay platforms (ELISAs), the U.S. SARS-CoV-2 standard is measured on the same 96-well plate as the daily assay standard, run as serial dilutions in triplicate and quadruplicate (Fig. 1). Standard curves are constructed for both the U.S. SARS-CoV-2 Serology standard and daily assay standard. A test of parallelism and linearity between the two dose-response curves is then performed to ensure that immunoaffinity differences or matrix effects do not prevent accurate calibration with the U.S. SARS-CoV-2 serology standard. Units based on the U.S. SARS-CoV-2 serology standard can then be assigned to the assay daily standard, to harmonize assays and units for result reporting. For non-plate-based assay platforms, similar dilution-based standard curves are constructed.

Traceability of the FNLCR standard to the first WHO international standard (IS) for anti-SARS-CoV-2 immunoglobulin (20/136) was established, to allow SeroNet assays to convert U.S. serology standard units to WHO IS units. WHO IS 20/136 is a freeze-dried equivalent of 0.25 mL of pooled plasma from 11 individuals with a history of SARS-CoV-2 infection. Once reconstituted, the WHO standard has an arbitrary unitage of 1,000 binding antibody units (BAU)/mL. Eight serial dilutions of the U.S. SARS-CoV-2 serology standard and WHO IS 20/136 were run in triplicate. Parallel line analysis, which included tests for parallelism and linearity, was utilized to assign WHO IS 20/136 standard units to the U.S. SARS-CoV-2 serology standard; this will allow SeroNet institutions to convert U.S. SARS-CoV-2 serology standard units to WHO standard units for serological methods.

### Patient consent statement.

This work involves a descriptive summary of serological assays and assay harmonization plans and does not include factors necessitating patient consent.

## References

[1] National Cancer Institute. 2022. NCI Serological Sciences Network for COVID-19 (SeroNet). https://www.cancer.gov/research/key-initiatives/covid-19/coronavirus-research-initiatives/serological-sciences-network. Accessed 2 December 2021.

[2] Food and Drug Administration (FDA). In vitro diagnostics EUAs—serology and other adaptive immune response tests for SARS-CoV-2. https://www.fda.gov/medical-devices/coronavirus-disease-2019-covid-19-emergency-use-authorizations-medical-devices/in-vitro-diagnostics-euas-serology-and-other-adaptive-immune-response-tests-sars-cov-2. Accessed 2 December 2021.

[3] Muecksch F, Wise H, Batchelor B, Squires M, Semple E, Richardson C, McGuire J, Clearly S, Furrie E, Greig N, Hay G, Templeton K, Lorenzi JCC, Hatziioannou T, Jenks S, Bieniasz PD. 2021. Longitudinal serological analysis and neutralizing antibody levels in coronavirus disease 2019 convalescent patients. J Infect Dis 223:389–398. doi:10.1093/infdis/jiaa659. 33140086 PMC7665595

[4] Perez-Saez J, Zaballa M-E, Yerly S, Andrey DO, Meyer B, Eckerle I, Balavoine J-F, Chappuis F, Pittet D, Trono D, Kherad O, Vuilleumier N, Kaiser L, Guessous I, Stringhini S, Azman AS, Specchio-COVID19 Study Group. 2021. Persistence of anti-SARS-CoV-2 antibodies: immunoassay heterogeneity and implications for serosurveillance. Clin Microbiol Infect 27:1695.e7–e1695.e12. doi:10.1016/j.cmi.2021.06.040. PMC8261139 34245905

[5] Muecksch F, Wise H, Templeton K, Batchelor B, Squires M, McCance K, Jarvis L, Malloy K, Furrie E, Richardson C, MacGuire J, Godber I, Burns A, Mavin S, Zhang F, Schmidt F, Bieniasz P, Jenks S, Hatziioannou T. 2021. Longitudinal variation in SARS-CoV-2 antibody levels and emergence of viral variants: implications for the ability of serological assays to predict immunity. medRxiv doi:10.1101/2021.07.02.21259939. PMC9141682 35636436

[6] Narowski TM, Raphel K, Adams LE, Huang J, Vielot NA, Jadi R, de Silva AM, Baric RS, Lafleur JE, Premkumar L. 2022. SARS-CoV-2 mRNA vaccine induces robust specific and cross-reactive IgG and unequal neutralizing antibodies in naive and previously infected people. Cell Rep 38:110336. doi:10.1016/j.celrep.2022.110336. 35090596 PMC8769879

[7] Sholukh AM, Fiore-Gartland A, Ford ES, Miner MD, Hou YJ, Tse LV, Kaiser H, Zhu H, Lu J, Madarampalli B, Park A, Lempp FA, St Germain R, Bossard EL, Kee JJ, Diem K, Stuart AB, Rupert PB, Brock C, Buerger M, Doll MK, Randhawa AK, Stamatatos L, Strong RK, McLaughlin C, Huang M-L, Jerome KR, Baric RS, Montefiori D, Corey L. 2021. Evaluation of cell-based and surrogate SARS-CoV-2 neutralization assays. J Clin Microbiol 59:e00527-21. doi:10.1128/JCM.00527-21. PMC8451402 34288726

[8] von Rhein C, Scholz T, Henss L, Kronstein-Wiedemann R, Schwarz T, Rodionov RN, Corman VM, Tonn T, Schnierle BS. 2021. Comparison of potency assays to assess SARS-CoV-2 neutralizing antibody capacity in COVID-19 convalescent plasma. J Virol Methods 288:114031. doi:10.1016/j.jviromet.2020.114031. 33275926 PMC7707675

[9] Olbrich L, Castelletti N, Schälte Y, Garí M, Pütz P, Bakuli A, Pritsch M, Kroidl I, Saathoff E, Guggenbuehl Noller JM, Fingerle V, Le Gleut R, Gilberg L, Brand I, Falk P, Markgraf A, Deák F, Riess F, Diefenbach M, Eser T, Weinauer F, Martin S, Quenzel EM, Becker M, Durner J, Girl P, Müller K, Radon K, Fuchs C, Wölfel R, Hasenauer J, Hoelscher M, Wieser A, on behalf of the KoCo-Study Group. 2021. Head-to-head evaluation of seven different seroassays including direct viral neutralisation in a representative cohort for SARS-CoV-2. J Gen Virol 102:001653. doi:10.1099/jgv.0.001653. PMC8604188 34623233

[10] Röltgen K, Powell AE, Wirz OF, Stevens BA, Hogan CA, Najeeb J, Hunter M, Wang H, Sahoo MK, Huang C, Yamamoto F, Manohar M, Manalac J, Otrelo-Cardoso AR, Pham TD, Rustagi A, Rogers AJ, Shah NH, Blish CA, Cochran JR, Jardetzky TS, Zehnder JL, Wang TT, Narasimhan B, Gombar S, Tibshirani R, Nadeau KC, Kim PS, Pinsky BA, Boyd SD. 2020. Defining the features and duration of antibody responses to SARS-CoV-2 infection associated with disease severity and outcome. Sci Immunol 5:54. doi:10.1126/sciimmunol.abe0240. PMC7857392 33288645

[11] Alter G, Yu J, Liu J, Chandrashekar A, Borducchi EN, Tostanoski LH, McMahan K, Jacob-Dolan C, Martinez DR, Chang A, Anioke T, Lifton M, Nkolola J, Stephenson KE, Atyeo C, Shin S, Fields P, Kaplan I, Robins H, Amanat F, Krammer F, Baric RS, Le Gars M, Sadoff J, de Groot AM, Heerwegh D, Struyf F, Douoguih M, van Hoof J, Schuitemaker H, Barouch DH. 2021. Immunogenicity of Ad26.COV2.S vaccine against SARS-CoV-2 variants in humans. Nature 596:268–272. doi:10.1038/s41586-021-03681-2. 34107529 PMC8357629

[12] Amanat F, Stadlbauer D, Strohmeier S, Nguyen THO, Chromikova V, McMahon M, Jiang K, Asthagiri Arunkumar G, Jurczyszak D, Polanco J, Bermudez-Gonzalez M, Kleiner G, Aydillo T, Miorin L, Fierer D, Amarilis Lugo L, Milunka Kojic E, Stoever J, Liu STH, Cunningham-Rundles C, Felgner PL, Moran T, Garcia-Sastre A, Caplivski D, Cheng A, Kedzierska K, Vapalahti O, Hepojoki JM, Simon V, Krammer F. 2020. A serological assay to detect SARS-CoV-2 seroconversion in humans. medRxiv doi:10.1101/2020.03.17.20037713. PMC8183627 32398876

[13] Arthur JM, Forrest JC, Boehme KW, Kennedy JL, Owens S, Herzog C, Liu J, Harville TO. 2021. Development of ACE2 autoantibodies after SARS-CoV-2 infection. PLoS One 16:e0257016. doi:10.1371/journal.pone.0257016. 34478478 PMC8415618

[14] Collier A-RY, McMahan K, Yu J, Tostanoski LH, Aguayo R, Ansel J, Chandrashekar A, Patel S, Apraku Bondzie E, Sellers D, Barrett J, Sanborn O, Wan H, Chang A, Anioke T, Nkolola J, Bradshaw C, Jacob-Dolan C, Feldman J, Gebre M, Borducchi EN, Liu J, Schmidt AG, Suscovich T, Linde C, Alter G, Hacker MR, Barouch DH. 2021. Immunogenicity of COVID-19 mRNA vaccines in pregnant and lactating women. JAMA 325:2370–2380. doi:10.1001/jama.2021.7563. 33983379 PMC8120446

[15] Klein SL, Pekosz A, Park H-S, Ursin RL, Shapiro JR, Benner SE, Littlefield K, Kumar S, Naik HM, Betenbaugh MJ, Shrestha R, Wu AA, Hughes RM, Burgess I, Caturegli P, Laeyendecker O, Quinn TC, Sullivan D, Shoham S, Redd AD, Bloch EM, Casadevall A, Tobian AA. 2020. Sex, age, and hospitalization drive antibody responses in a COVID-19 convalescent plasma donor population. J Clin Invest 130:6141–6150. doi:10.1172/JCI142004. 32764200 PMC7598041

[16] Premkumar L, Segovia-Chumbez B, Jadi R, Martinez DR, Raut R, Markmann A, Cornaby C, Bartelt L, Weiss S, Park Y, Edwards CE, Weimer E, Scherer EM, Rouphael N, Edupuganti S, Weiskopf D, Tse LV, Hou YJ, Margolis D, Sette A, Collins MH, Schmitz J, Baric RS, de Silva AM. 2020. The receptor binding domain of the viral spike protein is an immunodominant and highly specific target of antibodies in SARS-CoV-2 patients. Sci Immunol 5:eabc8413. doi:10.1126/sciimmunol.abc8413. 32527802 PMC7292505

[17] Salazar E, Kuchipudi SV, Christensen PA, Eagar T, Yi X, Zhao P, Jin Z, Long SW, Olsen RJ, Chen J, Castillo B, Leveque C, Towers D, Lavinder J, Gollihar J, Cardona J, Ippolito G, Nissly R, Bird I, Greenawalt D, Rossi RM, Gontu A, Srinivasan S, Poojary I, Cattadori IM, Hudson PJ, Josleyn NM, Prugar L, Huie K, Herbert A, Bernard DW, Dye JM, Kapur V, Musser JM. 2020. Convalescent plasma anti-SARS-CoV-2 spike protein ectodomain and receptor-binding domain IgG correlate with virus neutralization. J Clin Invest 130:6728–6738. doi:10.1172/JCI141206. 32910806 PMC7685744

[18] Seegmiller JC, Kokaisel EL, Story SJ, Zaun CP, Peters JM, Thomas SN, Karger AB. 2020. Method comparison of SARS-CoV-2 serology assays involving three commercially available platforms and a novel in-house developed enzyme-linked immunosorbent assay. Clin Biochem 86:34–35. doi:10.1016/j.clinbiochem.2020.08.004. 32791053 PMC7417259

[19] Stadlbauer D, Amanat F, Chromikova V, Jiang K, Strohmeier S, Arunkumar GA, Tan J, Bhavsar D, Capuano C, Kirkpatrick E, Meade P, Brito RN, Teo C, McMahon M, Simon V, Krammer F. 2020. SARS-CoV-2 seroconversion in humans: a detailed protocol for a serological assay, antigen production, and test setup. Curr Protoc Microbiol 57:e100. doi:10.1002/cpmc.100. 32302069 PMC7235504

[20] Stadlbauer D, Tan J, Jiang K, Hernandez MM, Fabre S, Amanat F, Teo C, Arunkumar GA, McMahon M, Capuano C, Twyman K, Jhang J, Nowak MD, Simon V, Sordillo EM, van Bakel H, Krammer F. 2021. Repeated cross-sectional sero-monitoring of SARS-CoV-2 in New York City. Nature 590:146–150. doi:10.1038/s41586-020-2912-6. 33142304

[21] Suthar MS, Zimmerman MG, Kauffman RC, Mantus G, Linderman SL, Hudson WH, Vanderheiden A, Nyhoff L, Davis CW, Adekunle O, Affer M, Sherman M, Reynolds S, Verkerke HP, Alter DN, Guarner J, Bryksin J, Horwath MC, Arthur CM, Saakadze N, Smith GH, Edupuganti S, Scherer EM, Hellmeister K, Cheng A, Morales JA, Neish AS, Stowell SR, Frank F, Ortlund E, Anderson EJ, Menachery VD, Rouphael N, Mehta AK, Stephens DS, Ahmed R, Roback JD, Wrammert J. 2020. Rapid generation of neutralizing antibody responses in COVID-19 patients. Cell Rep Med 1:100040. doi:10.1016/j.xcrm.2020.100040. 32835303 PMC7276302

[22] Thomas SN, Altawallbeh G, Zaun CP, Pape KA, Peters JM, Titcombe PJ, Dileepan T, Rapp MJ, Bold TD, Schacker TW, Arbefeville S, Ferrieri P, Thyagarajan B, Jenkins MK, Karger AB. 2021. Initial determination of COVID-19 seroprevalence among outpatients and healthcare workers in Minnesota using a novel SARS-CoV-2 total antibody ELISA. Clin Biochem 90:15–22. doi:10.1016/j.clinbiochem.2021.01.010. 33539808 PMC7849522

[23] Vidal SJ, Collier A-RY, Yu J, McMahan K, Tostanoski LH, Ventura JD, Aid M, Peter L, Jacob-Dolan C, Anioke T, Chang A, Wan H, Aguayo R, Ngo D, Gerszten RE, Seaman MS, Barouch DH. 2021. Correlates of neutralization against SARS-CoV-2 variants of concern by early pandemic sera. J Virol 95:e00404-21. doi:10.1128/JVI.00404-21. PMC8223959 33893169

[24] Zeng C, Evans JP, Pearson R, Qu P, Zheng Y-M, Robinson RT, Hall-Stoodley L, Yount J, Pannu S, Mallampalli RK, Saif L, Oltz E, Lozanski G, Liu S-L. 2020. Neutralizing antibody against SARS-CoV-2 spike in COVID-19 patients, health care workers, and convalescent plasma donors. JCI Insight 5:e143213. doi:10.1172/jci.insight.143213. PMC7710271 33035201

[25] Sariol C, Pantoja P, Serrano-Collazo C, Rosa-Arocho T, Armina-Rodríguez A, Cruz L, Stone E, Arana T, Climent C, Latoni G, Atehortua D, Pabon-Carrero C, Pinto A, Brien J, Espino A. 2021. Function is more reliable than quantity to follow up the humoral response to the receptor-binding domain of SARS-CoV-2-spike protein after natural infection or COVID-19 vaccination. Viruses 13:1972. doi:10.3390/v13101972. 34696403 PMC8538099

[26] Roy V, Fischinger S, Atyeo C, Slein M, Loos C, Balazs A, Luedemann C, Astudillo MG, Yang D, Wesemann DR, Charles R, Lafrate AJ, Feldman J, Hauser B, Caradonna T, Miller TE, Murali MR, Baden L, Nilles E, Ryan E, Lauffenburger D, Beltran WG, Alter G. 2020. SARS-CoV-2-specific ELISA development. J Immunol Methods 484–485:112832. doi:10.1016/j.jim.2020.112832. PMC7414735 32780998

[27] Rosenberg ES, Tesoriero JM, Rosenthal EM, Chung R, Barranco MA, Styer LM, Parker MM, John Leung S-Y, Morne JE, Greene D, Holtgrave DR, Hoefer D, Kumar J, Udo T, Hutton B, Zucker HA. 2020. Cumulative incidence and diagnosis of SARS-CoV-2 infection in New York. Ann Epidemiol 48:23–29.e4. doi:10.1016/j.annepidem.2020.06.004. 32648546 PMC7297691

[28] Styer LM, Hoen R, Rock J, Yauney E, Nemeth K, Bievenue R, Parker MM. 2021. High-throughput multiplex SARS-CoV-2 IgG microsphere immunoassay for dried blood spots: a public health strategy for enhanced serosurvey capacity. Microbiol Spectr 9:e00134-21. doi:10.1128/Spectrum.00134-21. PMC8552730 34319133

[29] Lee WT, Girardin RC, Dupuis AP, Kulas KE, Payne AF, Wong SJ, Arinsburg S, Nguyen FT, Mendu DR, Firpo-Betancourt A, Jhang J, Wajnberg A, Krammer F, Cordon-Cardo C, Amler S, Montecalvo M, Hutton B, Taylor J, McDonough KA. 2021. Neutralizing antibody responses in COVID-19 convalescent sera. J Infect Dis 223:47–55. doi:10.1093/infdis/jiaa673. 33104179 PMC7665673

[30] Yates JL, Ehrbar DJ, Hunt DT, Girardin RC, Dupuis AP, Payne AF, Sowizral M, Varney S, Kulas KE, Demarest VL, Howard KM, Carson K, Hales M, Ejemel M, Li Q, Wang Y, Peredo-Wende R, Ramani A, Singh G, Strle K, Mantis NJ, McDonough KA, Lee WT. 2021. Serological analysis reveals an imbalanced IgG subclass composition associated with COVID-19 disease severity. Cell Rep Med 2:100329. doi:10.1016/j.xcrm.2021.100329. 34151306 PMC8205277

[31] Pisanic N, Randad PR, Kruczynski K, Manabe YC, Thomas DL, Pekosz A, Klein SL, Betenbaugh MJ, Clarke WA, Laeyendecker O, Caturegli PP, Larman HB, Detrick B, Fairley JK, Sherman AC, Rouphael N, Edupuganti S, Granger DA, Granger SW, Collins MH, Heaney CD. 2020. COVID-19 serology at population scale: SARS-CoV-2-specific antibody responses in saliva. J Clin Microbiol 59:e02204-20. doi:10.1128/JCM.02204-20. 33067270 PMC7771435

[32] Canaday DH, Carias L, Oyebanji OA, Keresztesy D, Wilk D, Payne M, Aung H, St Denis K, Lam EC, Wilson B, Rowley CF, Berry SD, Cameron CM, Cameron MJ, Balazs AB, Gravenstein S, King CL. 2021. Reduced BNT162b2 mRNA vaccine response in SARS-CoV-2-naive nursing home residents. Clin Infect Dis 73:2112–2115. doi:10.1093/cid/ciab447. 33993265 PMC8240817

[33] Tian L, Elsheikh EB, Patrone PN, Kearsley AJ, Gaigalas AK, Inwood S, Lin-Gibson S, Esposito D, Wang L. 2021. Towards quantitative and standardized serological and neutralization assays for COVID-19. Int J Mol Sci 22:2723. doi:10.3390/ijms22052723. 33800363 PMC7962843

[34] Su Y, Chen D, Yuan D, Lausted C, Choi J, Dai CL, Voillet V, Duvvuri VR, Scherler K, Troisch P, Baloni P, Qin G, Smith B, Kornilov SA, Rostomily C, Xu A, Li J, Dong S, Rothchild A, Zhou J, Murray K, Edmark R, Hong S, Heath JE, Earls J, Zhang R, Xie J, Li S, Roper R, Jones L, Zhou Y, Rowen L, Liu R, Mackay S, O’Mahony DS, Dale CR, Wallick JA, Algren HA, Zager MA, Wei W, Price ND, Huang S, Subramanian N, Wang K, Magis AT, Hadlock JJ, Hood L, Aderem A, Bluestone JA, Lanier LL, ISB-Swedish COVID19 Biobanking Unit, et al. 2020. Multi-omics resolves a sharp disease-state shift between mild and moderate COVID-19. Cell 183:1479–1495.e20. doi:10.1016/j.cell.2020.10.037. 33171100 PMC7598382

[35] Xhangolli I, Dura B, Lee G, Kim D, Xiao Y, Fan R. 2019. Single-cell analysis of CAR-T cell activation reveals a mixed TH1/TH2 response independent of differentiation. Genomics Proteomics Bioinformatics 17:129–139. doi:10.1016/j.gpb.2019.03.002. 31229590 PMC6620429

[36] Heaney CD, Pisanic N, Randad PR, Kruczynski K, Howard T, Zhu X, Littlefield K, Patel EU, Shrestha R, Laeyendecker O, Shoham S, Sullivan D, Gebo K, Hanley D, Redd AD, Quinn TC, Casadevall A, Zenilman JM, Pekosz A, Bloch EM, Tobian AAR. 2021. Comparative performance of multiplex salivary and commercially available serologic assays to detect SARS-CoV-2 IgG and neutralization titers. J Clin Virol 145:104997. doi:10.1016/j.jcv.2021.104997. 34695724 PMC8502080

[37] Haddad NS, Nguyen DC, Kuruvilla ME, Morrison-Porter A, Anam F, Cashman KS, Ramonell RP, Kyu S, Saini AS, Cabrera-Mora M, Derrico A, Alter D, Roback JD, Horwath M, O'Keefe JB, Wu HM, Wong A-KI, Dretler AW, Gripaldo R, Lane AN, Wu H, Chu HY, Lee S, Hernandez M, Engineer V, Varghese J, Patel R, Jalal A, French V, Guysenov I, Lane CE, Mengistsu T, Normile KE, Mnzava O, Le S, Sanz I, Daiss JL, Lee FE-H. 2021. One-stop serum assay identifies COVID-19 disease severity and vaccination responses. Immunohorizons 5:322–335. doi:10.4049/immunohorizons.2100011. 34001652 PMC9190970

[38] Amanat F, White KM, Miorin L, Strohmeier S, McMahon M, Meade P, Liu W-C, Albrecht RA, Simon V, Martinez-Sobrido L, Moran T, García-Sastre A, Krammer F. 2020. An in vitro microneutralization assay for SARS-CoV-2 serology and drug screening. Curr Protoc Microbiol 58:e108. doi:10.1002/cpmc.108. 32585083 PMC7361222

[39] Wajnberg A, Amanat F, Firpo A, Altman DR, Bailey MJ, Mansour M, McMahon M, Meade P, Mendu DR, Muellers K, Stadlbauer D, Stone K, Strohmeier S, Simon V, Aberg J, Reich DL, Krammer F, Cordon-Cardo C. 2020. Robust neutralizing antibodies to SARS-CoV-2 infection persist for months. Science 370:1227–1230. doi:10.1126/science.abd7728. 33115920 PMC7810037

[40] Hassert M, Geerling E, Stone ET, Steffen TL, Feldman MS, Dickson AL, Class J, Richner JM, Brien JD, Pinto AK. 2020. mRNA induced expression of human angiotensin-converting enzyme 2 in mice for the study of the adaptive immune response to severe acute respiratory syndrome coronavirus 2. PLoS Pathog 16:e1009163. doi:10.1371/journal.ppat.1009163. 33326500 PMC7773324

[41] Vanderheiden A, Edara VV, Floyd K, Kauffman RC, Mantus G, Anderson E, Rouphael N, Edupuganti S, Shi P-Y, Menachery VD, Wrammert J, Suthar MS. 2020. Development of a rapid focus reduction neutralization test assay for measuring SARS-CoV-2 neutralizing antibodies. Curr Protoc Immunol 131:e116. doi:10.1002/cpim.116. 33215858 PMC7864545

[42] Girardin RC, Dupuis AP, Payne AF, Sullivan TJ, Strauss D, Parker MM, McDonough KA. 2021. Temporal analysis of serial donations reveals decrease in neutralizing capacity and justifies revised qualifying criteria for coronavirus disease 2019 convalescent plasma. J Infect Dis 223:743–751. doi:10.1093/infdis/jiaa803. 33417696 PMC7928872

[43] Dhakal S, Ruiz-Bedoya CA, Zhou R, Creisher PS, Villano JS, Littlefield K, Ruelas Castillo J, Marinho P, Jedlicka AE, Ordonez AA, Bahr M, Majewska N, Betenbaugh MJ, Flavahan K, Mueller ARL, Looney MM, Quijada D, Mota F, Beck SE, Brockhurst J, Braxton AM, Castell N, Stover M, D’Alessio FR, Metcalf Pate KA, Karakousis PC, Mankowski JL, Pekosz A, Jain SK, Klein SL. 2021. Sex differences in lung imaging and SARS-CoV-2 antibody responses in a COVID-19 golden Syrian hamster model. mBio 12:e00974-21. doi:10.1128/mBio.00974-21. PMC8406232 34253053

[44] Morgenlander WR, Henson SN, Monaco DR, Chen A, Littlefield K, Bloch EM, Fujimura E, Ruczinski I, Crowley AR, Natarajan H, Butler SE, Weiner JA, Li MZ, Bonny TS, Benner SE, Balagopal A, Sullivan D, Shoham S, Quinn TC, Eshleman SH, Casadevall A, Redd AD, Laeyendecker O, Ackerman ME, Pekosz A, Elledge SJ, Robinson M, Tobian AA, Larman HB. 2021. Antibody responses to endemic coronaviruses modulate COVID-19 convalescent plasma functionality. J Clin Invest 131:e146927. doi:10.1172/JCI146927. PMC8011893 33571169

[45] Ogega CO, Skinner NE, Blair PW, Park HS, Littlefield K, Ganesan A, Dhakal S, Ladiwala P, Antar AA, Ray SC, Betenbaugh MJ, Pekosz A, Klein SL, Manabe YC, Cox AL, Bailey JR. 2021. Durable SARS-CoV-2 B cell immunity after mild or severe disease. J Clin Invest 131:e145516. doi:10.1172/JCI145516. PMC8011891 33571162

[46] Kared H, Redd AD, Bloch EM, Bonny TS, Sumatoh H, Kairi F, Carbajo D, Abel B, Newell EW, Bettinotti MP, Benner SE, Patel EU, Littlefield K, Laeyendecker O, Shoham S, Sullivan D, Casadevall A, Pekosz A, Nardin A, Fehlings M, Tobian AA, Quinn TC. 2021. SARS-CoV-2-specific CD8+ T cell responses in convalescent COVID-19 individuals. J Clin Invest 131:e145476. doi:10.1172/JCI145476. PMC7919723 33427749

[47] Patel EU, Bloch EM, Clarke W, Hsieh Y-H, Boon D, Eby Y, Fernandez RE, Baker OR, Keruly M, Kirby CS, Klock E, Littlefield K, Miller J, Schmidt HA, Sullivan P, Piwowar-Manning E, Shrestha R, Redd AD, Rothman RE, Sullivan D, Shoham S, Casadevall A, Quinn TC, Pekosz A, Tobian AAR, Laeyendecker O. 2021. Comparative performance of five commercially available serologic assays to detect antibodies to SARS-CoV-2 and identify individuals with high neutralizing titers. J Clin Microbiol 59:e02257-20. doi:10.1128/JCM.02257-20. 33139419 PMC8111143

[48] Röltgen K, Nielsen SCA, Arunachalam PS, Yang F, Hoh RA, Wirz OF, Lee AS, Gao F, Mallajosyula V, Li C, Haraguchi E, Shoura MJ, Wilbur JL, Wohlstadter JN, Davis MM, Pinsky BA, Sigal GB, Pulendran B, Nadeau KC, Boyd SD. 2021. mRNA vaccination compared to infection elicits an IgG-predominant response with greater SARS-CoV-2 specificity and similar decrease in variant spike recognition. medRxiv doi:10.1101/2021.04.05.21254952.

[49] Pegu A, O’Connell S, Schmidt SD, O’Dell S, Talana CA, Lai L, Albert J, Anderson E, Bennett H, Corbett KS, Flach B, Jackson L, Leav B, Ledgerwood JE, Luke CJ, Makowski M, Roberts PC, Roederer M, Rebolledo PA, Rostad CA, Rouphael NG, Shi W, Wang L, Widge AT, Yang ES, mRNA-1273 Study Group, Beigel JH, Graham BS, Mascola JR, Suthar MS, McDermott A, Doria-Rose NA. 2021. Durability of mRNA-1273-induced antibodies against SARS-CoV-2 variants. bioRxiv doi:10.1101/2021.05.13.444010. PMC8691522 34385356

[50] Weissman D, Alameh M-G, de Silva T, Collini P, Hornsby H, Brown R, LaBranche CC, Edwards RJ, Sutherland L, Santra S, Mansouri K, Gobeil S, McDanal C, Pardi N, Hengartner N, Lin PJC, Tam Y, Shaw PA, Lewis MG, Boesler C, Şahin U, Acharya P, Haynes BF, Korber B, Montefiori DC. 2021. D614G spike mutation increases SARS CoV-2 susceptibility to neutralization. Cell Host Microbe 29:23–31.e4. doi:10.1016/j.chom.2020.11.012. 33306985 PMC7707640

[51] Figueiredo JC, Hirsch FR, Kushi LH, Nembhard WN, Crawford JM, Mantis N, Finster L, Merin NM, Merchant A, Reckamp KL, Melmed GY, Braun J, McGovern D, Parekh S, Corley DA, Zohoori N, Amick BC, Du R, Gregersen PK, Diamond B, Taioli E, Sariol C, Espino A, Weiskopf D, Gifoni A, Brien J, Hanege W, Lipsitch M, Zidar DA, McAlearney AS, Wajnberg A, LaBaer J, Lewis EY, Binder RA, Moormann AM, Forconi C, Forrester S, Batista J, Schieffelin J, Kim D, Biancon G, VanOudenhove J, Halene S, Fan R, Barouch DH, Alter G, Pinninti S, Boppana SB, Pati SK, Latting M, et al. 27 April 2022. Mission, organization and future direction of the Serological Sciences Network for COVID-19 (SeroNet) Epidemiologic Cohort Studies. Open Forum Infect Dis doi:10.1093/ofid/ofac171. PMC9129196 35765315

[52] WHO. 2022. WHO manual for the preparation of reference materials for use as secondary standards in antibody testing. https://www.who.int/publications/m/item/who-manual-for-reference-material-for-antibody-testing. Accessed 20 May 2022.

[53] Espino AM, Pantoja P, Sariol CA. 2020. Validation and performance of a quantitative IgG assay for the screening of SARS-CoV-2 antibodies. bioRxiv 2020.06.11.146332. doi:10.1101/2020.06.11.146332.

[54] Boehme KW, Kennedy JL, Snowden J, Owens SM, Kouassi M, Mann RL, Paredes A, Putt C, James L, Jin J, Du R, Kirkpatrick C, Modi Z, Caid K, Zohoori N, Kothari A, Boyanton BL, Jr, Forrest JC. 2021. Pediatric SARS-CoV-2 seroprevalence in Arkansas over the first year of the COVID-19 pandemic. medRxiv 2021.08.04.21261592. doi:10.1101/2021.08.04.21261592. PMC8992271 35294550

